# The impact of obesity and smoking on young individuals suffering from lumbar disc herniation: a retrospective analysis of 97 cases

**DOI:** 10.1007/s10143-019-01151-y

**Published:** 2019-08-14

**Authors:** Sara Lener, Christoph Wipplinger, Sebastian Hartmann, Claudius Thomé, Anja Tschugg

**Affiliations:** Department of Neurosurgery, Medical University of Innsbruck, Innsbruck, Austria

**Keywords:** Obesity, Smoking, Young adults, Adolescent, Lumbar disc herniation

## Abstract

The negative impact on spinal diseases may apply not only to obesity but also to smoking. To investigate the influence of obesity and smoking on the development and recovery of lumbar disc herniation in young adults. Retrospective analysis of 97 patients who presented with lumbar disc herniation at the authors’ department between 2010 and 2017. Data were collected using the patients’ digital health records including demographics, clinical and neurological characteristics, treatment details, and outcomes. Ninety-seven patients between 17 and 25 years were included in this retrospective analysis. Patients were categorized into two groups according to their body mass index: obese (O, ≥ 30 kg/m^2^) and non-obese (NO, < 30 kg/m^2^). The proportion of obese patients in our cohort vs. in the overall population differed significantly (19.4% vs. 3.8–7.1%, RR 3.17; *p* < 0.01). Group NO showed a trend toward faster recovery of motor deficits (*p* = 0.067) and pain (*p* = 0.074). Also, the proportion of regular smokers differed significantly from the numbers of known smokers of the same age (62.4% vs. 30.2%, RR 2.0; *p* = 0.01). Obesity plus smoking showed a significantly negative impact on motor deficits postoperatively (*p* = 0.015) and at discharge (*p* = 0.025), as well as on pain values (*p* = 0.037) and on analgesic consumption (*p* = 0.034) at 6 weeks follow-up. The negative impact of obesity and smoking on the occurrence of lumbar disc herniation could be demonstrated for individuals aged 25 or younger. Furthermore, a trend to earlier recovery of motor deficits and significantly lower pain scales for non-obese and non-smoking patients could be shown.

## Introduction

Obesity is a growing pervasive disease in developing countries, and its prevalence might rise in future decades, especially in young adults [1]. Currently, obesity affects 3.8–7.1% of young adults in Central Europe [2, 3] and is defined as a body mass index (BMI) above 30 kg/m^2^. The impact of obesity on spinal and musculoskeletal disease (i.e., lower back pain, facet joint degeneration, and intervertebral disc degeneration) has been demonstrated in numerous previous studies [4, 5]. Influences were also detected for juvenile disc degeneration [6]. However, data on the impact of obesity on the treatment of lumbar disc herniation (LDH) for adolescents is lacking. Although an increased perioperative risk could be demonstrated in previous trials [7, 8], spinal surgery is presented as the treatment of choice with favorable outcomes in obese patients [9]. The management of LDH may be more challenging in young individuals since surgical indications are restricted, even though operative treatment shows a good outcome in adolescents [10, 11]. The outcome after lumbar sequesterectomy may be influenced by various other factors including gender and preoperative lifestyle habits [12, 13]. In addition to obesity, smoking also represents an important factor that influences the health of young individuals, as nearly one-third of people aged 30 and younger in Central Europe are regular smokers [14]. Smoking habits were found to have a negative impact not only on the development of LDH [15, 16], but also on re-herniation rates [17]. Nevertheless, the impact of smoking on recovery, especially in adolescents, is not clear. Also, the question whether an elevated BMI precedes or follows first clinical manifestations could not be answered yet. Therefore, the purpose of this retrospective study was to detect differences between obese and non-obese, and smoking and non-smoking young adults suffering from LDH, in terms of clinical features (i.e., time of diagnosis, motor deficits, pain scales, treatment details) and outcome.

## Material and methods

A retrospective analysis of patients aged between 17 and 25 years (22.3 ± 2.2) treated with sequestrectomy for LDH between 2010 and 2017 at the author’s department was done. Patients were categorized into two groups according to their BMI: group obese (O, ≥ 30 kg/m^2^) and group non-obese (NO, < 30 kg/m^2^). Additionally, three subgroups were created: group O1, obese and heavy smoking (≥ 20 cigarettes per day); group O2, obese and smoking (< 20 cigarettes per day); group O3, obese + non-smoking. These groups (consisting of 6 patients each) were analyzed separately. A subgroup analysis of smoking/non-smoking non-obese patients was not done concerning this study, as it is part of a separate investigation, dealing with the smoking status only.

The diagnosis was based on clinical and magnetic resonance imaging (MRI) parameters. Due to the patients’ young age, computed tomography (CT) was renounced. Patients were treated according to the guidelines of the German Society of Neurosurgery (DGNC) and the German Society of Orthopedics and Orthopedic Surgery (DGOOC) [18], where surgical treatment is considered if (1) the patient is unresponsive to maximum conservative treatment for at least 6 weeks, (2) shows progression of clinical symptoms, or (3) shows acute motor deficits, i.e., muscle force ≤ 3/5 according to Medical Research Council (MRC) scales. Data were collected using the patients’ digital health records (Cerner Millennium – Power Chart, Cerner Corporation 2011, Idstein, Germany) including demographics, baseline clinical and neurological characteristics, treatment details, and treatment outcomes of the patients. Pain values were assessed by Numeric Rating Scale (NRS) scores, “0” indicating “no pain” and “10” indicating “unbearable pain.” American Association of Anesthesiologists (ASA) scores were evaluated preoperatively for classification of the general health status of each patient, reaching from score “1” (normal, healthy patient) to “5” (multimorbid patient who will not survive without surgery). These parameters were gathered at four different time points: the initial diagnosis, the third postoperative day, the day of discharge, and 6 weeks postoperatively. Data were documented according to institutional standards and the general standards according to the principles of good clinical practice (GCP). Due to inconsistency of long-term follow-up data, special attention was paid to short-term follow-up. Clinical outcome parameters assessed in this study included the occurrence of motor deficits pre- and postoperatively and at 6 weeks follow-up, as well as pain scales and consumption of pain killers at the exact same time points.

### Patient cohort

Ninety-seven patients were identified and their data were analyzed retrospectively. All patients treated for lumbar disc herniation between 2010 and 2017 aged 25 or younger were considered for inclusion in our analysis. Four patients, all treated conservatively, all non-obese, were excluded, as follow-up organization in medically treated LDH is not standardized in our department and was less than 6 weeks in those cases. The main comparison in this analysis was performed between the obese and smoking groups (O1 + O2) and the remaining patients (O3 + NO).

The proportion of obese individuals (group O, *n* = 18) to non-obese individuals (group NO, *n* = 75) was 19.4% and 80.6%. This rate of obese individuals in our cohort (19.4%) differs significantly from the rate of obese adolescents in the Central European overall population (3.8–7.1%, RR 3.17, CI 1.32–7.6; *p* < 0.001) [2, 3]. Fifty-eight (62.4%) of all 130 patients were smokers. In addition, this rate shows a significant variation from the known percentage of smoking adolescents aged younger than 30 (30.2%, RR 2.0, CI 1.44–2.78; *p* = 0.01) [14]. The number of cigarettes per day was not significantly different between the two groups (*p* = 0.41; see Table 1). In total, 44.1% (*n* = 41) of patients were female. The distinction of the distribution of ASA scores was significant between the two groups with better overall scores in the non-obese patient group. The most frequently affected disc level was L5/S1 (*n* = 50, 53.8%) followed by L4/5 (*n* = 35, 37.6%). Level L1/2 was treated one time (*n* = 1, 1.1%), level L3/4 was affected two times (*n* = 2, 2.2%), and “other” levels, meaning L5/6 in the appearance of a sacral transitional vertebra, were treated four times altogether (*n* = 4, 4.3%). Lumbar sequestrectomy was performed as a standard in all patients (*n* = 93, 100%). A translaminar approach was chosen in two patients (2.7%), while the majority of patients was treated via an interlaminar approach (*n* = 73, 97.3%). None of the patients experienced intra- or perioperative complications (i.e., hematoma, infection, cerebrospinal fluid leakage) (Table 1).

**Table 1:** Demographic details

		Group O (*n* = 18)	Group NO (*n* = 75)	
Age	In years *(SD)*	21.3 (± 1.5)	22.2 (± 2.3)	n.s.
Sex, *n* (%)	Male	8 (44.4)	44 (58.7)	n.s.
	Female	10 (55.6)	31 (41.3)	n.s.
BMI	In kg/m^2^ (SD)	32.8 (± 3.5)	23.8 (± 2.8)	*p < 0.01*
ASA score, *n* (%)	°1	4 (22.2)	69 (92.0)	*p < 0.01*
	°2	14 (77.8)	6 (8.0)	*p < 0.01*
Smoking, *n* (%)		12 (66.7)	46 (61.3)	n.s.
	Cigarettes/day (SD)	10.3 (± 8.6)	8.8 (± 9.3)	n.s.
Duration of symptoms	In days (SD)	95.1 (± 76.0)	104.0 (± 76.4)	n.s.
Duration of hospital stay	In days (SD)	7.1 (± 2.1)	6.0 (± 2.4)	*p < 0.05*
Level of disc herniation, *n* (%)	L1/2	1 (5.6)	0	n.s.
	L3/4	1 (5.6)	1 (1.3)	n.s.
	L4/5	5 (27.8)	30 (40.0)	n.s.
	L5/S1	11 (61.2)	40 (53.3)	n.s.
	Other	0	4 (5.3)	n.s.
Operative time		84.4 (± 31.6)	68.8 (± 29.8)	n.s.
Complications	In minutes (SD)	0	0	n.s.

*p* values were calculated by comparison of the mean values of group O and group NO

*n* number of patients; *n*.*s* not significant; *SD* standard deviation

### Surgical procedure

Surgery was performed under the same standardized surgical protocol by different trial designated surgeons. After induction of general endotracheal anesthesia and with the assistance of an operating microscope, a microsurgical sequesterectomy was performed, while the patient was in a prone position. In cases of non-dislocated, non-cranially herniated discs, the spinal canal was exposed by performing a standard interlaminar fenestration. A translaminar approach was preferred for cranially herniated discs [19]. Based on previous trials, only the herniated disc material was removed, and, whenever possible, the annulus defect was not entered [20]. Intraoperative and postoperative complications like revision surgery for re-herniation, infection, or hematoma were recorded.

### Statistical analysis

All patients with complete initial data were considered for inclusion in this retrospective analysis. All values are expressed as mean ± standard deviation (SD). The Kolmogorov–Smirnov test was used for testing normal distribution. The unpaired Student’s *t* test and Mann–Whitney *U* test were performed to analyze differences in clinical and demographic characteristics and in clinical outcome variables. Frequencies were compared by chi-square and Fisher’s exact tests. Spearman’s rho correlation (*r*) was determined to assess the relationship between clinical outcome and demographic findings. A multivariate analysis of variance was performed to identify the coherence of combined findings and the clinical outcome. Relative risk ratio (RR) and 95% confidence intervals (CI) were calculated for the particular proportion of obese and smoking individuals. The level of significance was set to *p* < 0.05. All statistical evaluations were performed with SPSS Version 21.0 (IBM Corp., released 2012, IBM SPSS Statistics for Mac OS X, Version 21.0, NY, IBM Corp.). Figures were designed using Microsoft Excel (Version 15.36 for Mac OS X, Microsoft Corporation 2017, Redmond, USA).

## Results

The average duration of symptoms was 95 ± 76 days in group O vs. 104 ± 76 days in group NO (*p* = 0.59). The mean length of hospital stay showed a significant difference between group O (7 days ± 2) and group NO (6 days ± 2; *p* = 0.01). Operative time showed a trend to longer surgery in group O (O 84 ± 31 min vs. NO 69 ± 29 min; *p* = 0.06).

**Fig. 1:**
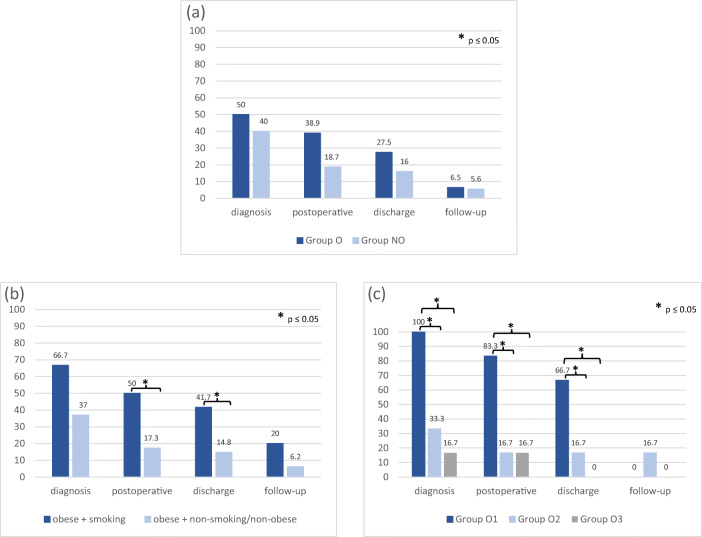
Differences in the incidence of motor deficits (percent, *y*-axis): between group O and group NO (**a**), between obese plus smoking patients versus obese non-smoking/non-obese patients (**b**), and among group O1 versus groups O2 and O3 at four different time points (*x*-axis) (**c**). Significant differences (*p* < 0.05) are marked by an asterisk

**Fig. 2:**
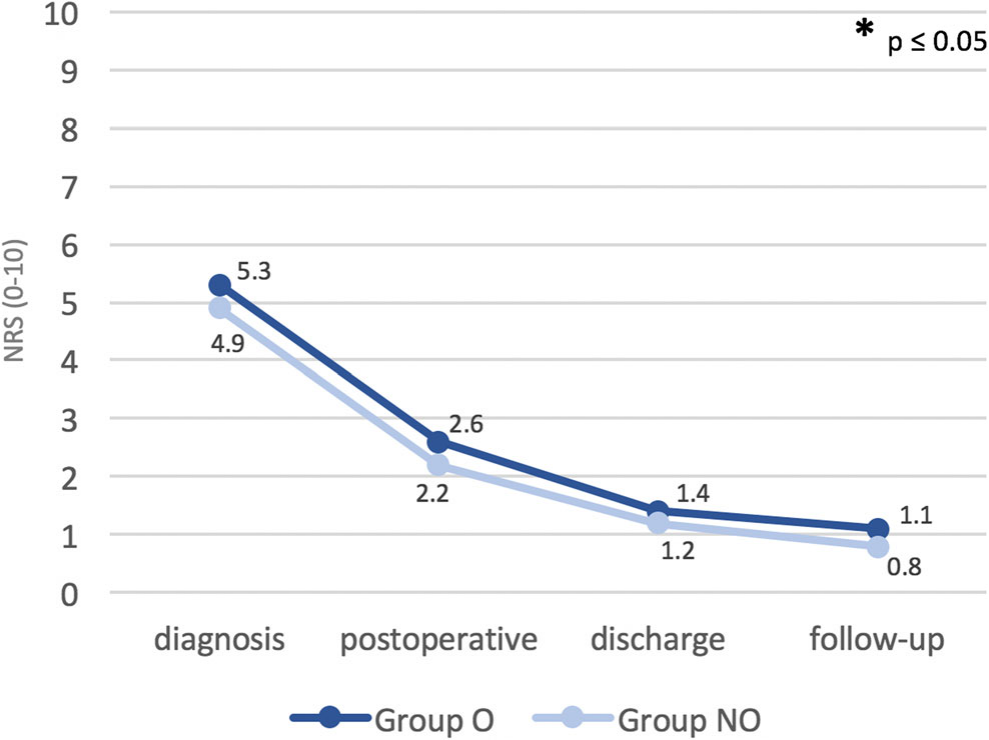
Differences in pain values, assessed by the NRS among group O and group NO at four different time points (*x*-axis). Significant differences (*p* < 0.05) are marked by an asterisk

Among the collected clinical outcome parameters, no significant differences between the two groups (O and NO) could be revealed for the extent of pain or motor deficit. Nevertheless, the data showed a trend to earlier recovery of motor deficits (*p* = 0.067) and pain scales in group NO (*p* = 0.074; see Figs. 1 and 2). Results were comparable for both groups 6 weeks postoperatively (*p* > 0.05).

For a subgroup analysis (groups O1, O2, and O3), significant alternations could be found comparing the groups with each other, and also in correlation to the rest of our population (group NO). The duration of hospital stay was significantly longer in groups O1 and O2, when compared with groups O3 and NO (6 ± 3 days vs. 7 ± 2 days; *p* = 0.04). Obese smoking patients showed a trend of a higher incidence of motor deficits at diagnosis (*n* = 8/12, 66.7% vs. *n* = 30/81, 37.0%; *p* = 0.063), and recovered significantly delayed, as seen in the rates of deficits 3 days postoperatively (*n* = 6/12, 50% vs. *n* = 14/81, 17.3%; *p* = 0.015) and at discharge (*n* = 5/12, 41.7% vs. *n* = 12/81, 14.8%; *p* = 0.025). After 6 weeks, the differences resolved (*n* = 1/12, 20% vs. *n* = 5/81, 6.2%; *p* = 0.69) (Fig. 1). No differences existed 6 weeks postoperatively (*n* = 0/6, 0% vs. *n* = 0/6, 0%).

**Fig. 3:**
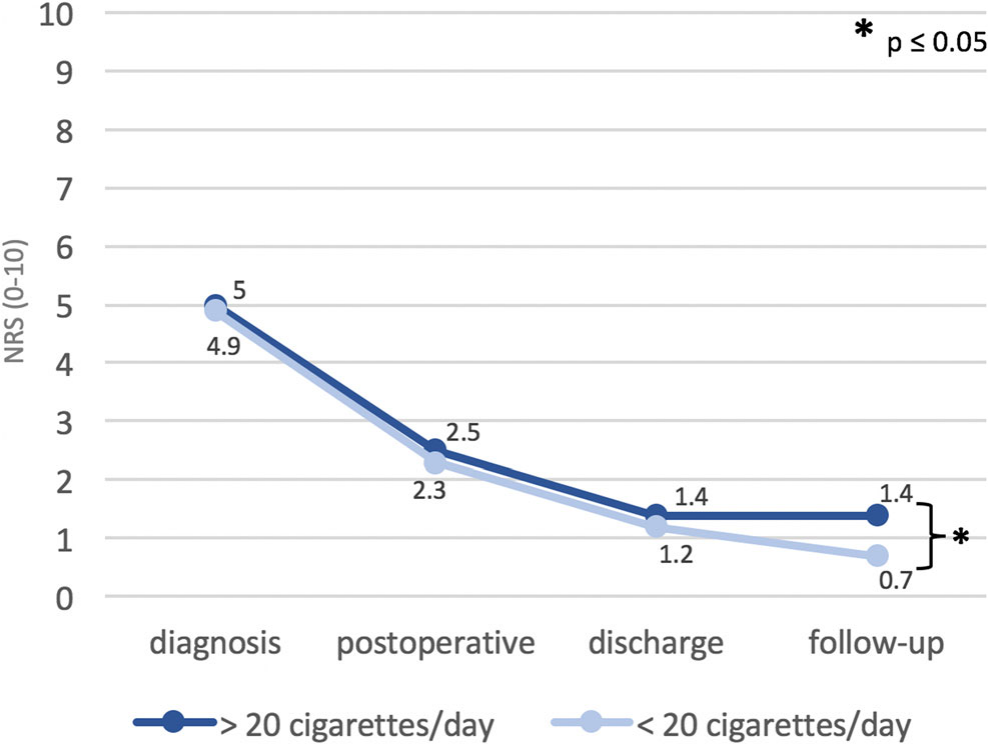
Differences in pain values, assessed by the NRS (0–10, *y*-axis) among smoking and non-smoking patients at four different time points (*x*-axis). Significant differences (*p* < 0.05) are marked by an asterisk

Moreover, non-obese patients’ pain outcomes at the 6 weeks follow-up were affected negatively by excessive smoking habits. Higher pain values (0.7 ± 1.1 vs. 1.4 ± 1.3; *p* = 0.026), as well as the usage of analgesics (*n* = 2/60, 3% vs. *n* = 4/17, 24%; *p* = 0.015), correlated with the consumption of > 20 cigarettes per day (Fig. 3).

Differences in the recovery of motor deficits maintained significant in obese patients with a consumption of > 20 cigarettes per day (group O1). Values were compared with those of obese patients smoking 20 cigarettes or fewer (group O2), and to obese, non-smoking patients (group O3; see Fig. 2). Group O1 showed significantly more motor deficits in comparison with group O3 at initial diagnosis (*n* = 6/6, 100% vs. *n* = 1/6, 16.7%; *p* = 0.020), on the third postoperative day (*n* = 5/6, 83.3% vs. *n* = 1/6, 16.7%; *p* = 0.021) and at discharge (*n* = 4/6, 66.7% vs. *n* = 0/6; 0%; *p* = 0.014). Group O1 vs. group O2 also showed significant differences in favor of group O2: motor deficits at diagnosis (*n* = 6/6, 100% vs. *n* = 2/6, 33.3%; *p* = 0.014) and on the third postoperative day (*n* = 5/6, 83.3%, vs. *n* = 1/6, 16.7%; *p* = 0.021) were more frequent among group O1. The differences remained at the day of discharge and at 6 weeks follow-up, but failed to reach significance (*n* = 4/6, 66.7% vs. *n* = 1/6, 16.7%; *p* = 0.079; *n* = 0/6, 0% vs. *n* = 1/6, 16.7%; *p* = 0.29, respectively).

A multivariate analysis revealed that the combination of overweight and heavy smoking resulted not only in a slower recovery of motor deficits (*p* = 0.07) but also in significantly higher pain values (*p* = 0.037), and therefore in a significantly more frequent usage of analgesics at 6 weeks follow-up (*p* = 0.004).

No gender differences were found in the presentation and outcome of lumbar disc herniation.

## Discussion

We report the results of the first retrospective study investigating the influence of obesity and smoking on individuals aged 25 years or less, suffering from LDH requiring treatment.

In total, 19.4% of treated patients presented with a BMI ≥ 30 kg/m^2^, whereby the overall prevalence for obesity at the investigated age level ranges from 3.8 to 7.1% in Central Europe [2, 3]. This reveals a significantly higher rate of obese individuals among our cohort and may demonstrate the impact of obesity on the genesis of lumbar disc herniations among young individuals. These findings agree with previous studies among older patients, which identified obesity as a significant risk factor for the development of spinal diseases, including intervertebral disc degeneration [4, 21, 22]. Nevertheless, the question whether an elevated BMI precedes or follows first clinical manifestations could not be answered, as beginning symptoms may lead to lower levels of physical activity and thus result in higher levels of body weight [6]. In our cohort, obese patients were solely treated surgically [9, 22], although higher perioperative risks have been described [8]. The shorter mean duration of symptoms until treatment starts in obese patients could assume that obese patients are (subjectively) more disabled by symptoms [23]. Furthermore, pain therapy is challenging, not only due to differences in drug metabolism [24, 25] that may consequently lead to earlier and more aggressive treatments. Nevertheless, none of our investigated patients experienced considerable perioperative complications. Thus, the hypothesis of higher risks concerning surgical intervention [7, 8] in obese patients could not be confirmed for patients aged 25 years or younger. Still, the fact that patients presenting with obesity showed significantly longer hospital stays cannot be neglected and may support the theory of delayed recovery.

Differently, the improvement of preoperative motor deficits depends on various factors like the duration of symptoms and on BMI [26]: the obese patient group in our cohort experienced symptoms for a shorter period but needed the same amount of time to recover from motor deficits as non-obese patients. Therefore, the overall factor of time and its impact on short-term recovery may be neglected for young patients presenting with obesity. Yet, the finding of the impact of time on motor deficit, its outcome, and on long-term recovery may not be valid as both factors could not be tested precisely enough for our study cohort. In general, patients with herniation of a lumbar disc, which causes mild or severe weakness, show a complete or almost complete recovery of strength after surgery [27]. In our patient cohort, the direct correlation between surgery and conservative treatment was not done, due to a missing medically treated population. For the general population, the 1-year outcomes were similar for patients assigned to early surgery and those assigned to conservative treatment [28]. Nevertheless, the rates of pain relief and of perceived recovery were faster for those assigned to early surgery. However, the circumstance of a higher BMI leading to a poorer neurological outcome could be proven to some extent.

Not only does the number of obese individuals in our cohort differ significantly from the known rate among the European population, but also the number of regular smokers was significantly higher in our cohort than known in adolescents aged 30 or younger (62.4% vs. 30.2%). Also, this finding may demonstrate the negative impact of regular smoking, not only on the recovery process but also on the development of lumbar disc disease in young individuals. Smoking has already been identified as a risk factor for the development of LDH [15, 16] as well as for the risk of re-herniation [17]. Likewise, the negative impact of smoking on the improvement of the functional status is already known [29]. Nevertheless, those factors were not tested in young individuals, as the recovery of pain especially seems to be negatively affected by heavy smoking, reaching a level of significance at 6 weeks of follow-up.

The risk of suffering from a motor deficit and experiencing a worse outcome or a delayed improvement rises for obese and smoking patients. Differences in impairment and postoperative deficits increase with rising numbers of cigarettes per day. When compared with obese non-smoking patients, the group presenting with obesity plus smoking behavior initially suffered from motor deficits significantly more often. Differences were sustained at 3 days postoperatively, but nearly adjusted at discharge and continued to improve until 6 weeks postoperatively. Incidence and adjustment rates worsened when obese patients were smoking > 20 cigarettes per day. We might confirm a negative effect of obesity plus smoking on the incidence of motor deficits in young patients. The same negative effect could be shown on the improvement of motor deficits, but only for a short period of time. Nevertheless, the impact of smoking not only on motor recovery but also on pain improvement cannot be ignored. Heavily smoking patients showed significantly worse pain outcomes, as well as a significantly more frequent use of analgesic medication after 6 weeks of follow-up.

Underlying mechanisms for a delay in motor deficit regeneration may include the chronic inflammatory conditions in our patient group, caused by both smoking and obesity [30–32]. Previous studies suggest an influence of high levels of cytokines not only on disc degeneration [33] but also on motor recovery and pain [34, 35]. Long-time consequences could not be determined properly in this retrospective setting. Nevertheless, causes can only be assumed and definitely need further investigation by prospective clinical trials.

Our retrospective results support the hypothesis that smoking is associated with poorer outcome after treatment for LDH, especially in the presence of obesity. Nevertheless, those results seem to have limited applicability for young patients in general, as individuals in both groups show similar outcomes after 6 weeks of follow-up. However, several limitations have to be considered when interpreting our results, including the retrospective study design, the different surgeons performing surgeries, the relatively small number of patients, and the short period of follow-up, as re-herniation rates and subjective long-term satisfaction could not be assessed. All patients were treated surgically and therefore the aspects of a conservative treatment in obese young individuals are mostly missing.

## Conclusion

We could demonstrate the negative impact of obesity and smoking on occurrence of LDH, but not precisely on the recovery of radiculopathy in young adults. Hence, when combined with heavy smoking, delayed recovery also applies to individuals aged 25 or younger and even isolated smoking may increase the risk to develop a LDH in individuals aged 30 or less.
